# Evaluation of Antileishmanial Activity of Albaha Medicinal Plants against *Leishmania amazonensis*

**DOI:** 10.1155/2015/938747

**Published:** 2015-08-19

**Authors:** Saeed S. Al-Sokari, Nasser A. Awadh Ali, Lianet Monzote, Mohamed A. Al-Fatimi

**Affiliations:** ^1^Department of Biology, Faculty of Sciences, Al Baha University, Saudi Arabia; ^2^Pharmacognosy Department, Faculty of Clinical Pharmacy, Al Baha University, Saudi Arabia; ^3^Departamento de Parasitología, Instituto de Medicina Tropical Pedro Kourí, La Habana, Cuba; ^4^Pharmacognosy Department, Faculty of Pharmacy, Aden University, P.O. Box 5411, Ma'alla, Aden, Yemen

## Abstract

Sixteen methanolic extracts obtained from thirteen plant species, selected either from ethnobotanical or chemotaxonomical data, were screened for their antileishmanial activity against *Leishmania amazonensis*. The cytotoxic activity against normal peritoneal macrophages from normal BALB/c mice was also determined. Eight extracts had IC_50_ values ranging from <12.5 to 37.8 *µ*g/mL against promastigotes. *Achillea biebersteinii* flower, *Euphorbia helioscopia,* and *Solanum incanum* leaf extracts showed antileishmanial activities with IC_50_ between <12.5–26.9 *µ*g/mL and acceptable selectivity indices of 8–5. The other leishmanicidal plant extracts, with IC_50_ ranging from 18.0 to 29.5 *µ*g/mL, exhibited low selectivity indices.

## 1. Introduction

Leishmaniasis is distributed worldwide, especially in tropical and subtropical areas and more than 12 million people are currently infected. About 20 000 to 30 000 deaths occur annually. Cutaneous leishmaniasis (CL) is the most common form of leishmaniasis and causes skin lesions, mainly ulcers. About 95% of CL cases occur in the Americas, the Mediterranean basin, the Middle East, and Central Asia. Over two-thirds of new CL cases occur in 6 countries: Afghanistan, Algeria, Brazil, Colombia, Iran, and Syria. An estimated 0.7 million to 1.3 million new cases occur worldwide annually [1, 2].

In Saudi Arabia, the disease was first described in 1973 by Morsy and Shoura [3]. Currently, CL is common in the human population in different localities, including the Eastern Province of Saudi Arabia and in particular the Al-Hassa Oasis that is a known endemic area for CL [4]. At the moment, no good conventional treatments for CL in most countries are available. The conventional drugs are very toxic and not 100% effective. Therefore, there is an urgent need to discover new therapeutic agents. Traditional medicine knowledge can be useful to open new ways in the search of new antileishmanial agents. A number of published studies have demonstrated that many plant extracts exhibited activity against* Leishmania* parasites [5–8]. Some reports were published about the antiprotozoal activity of Saudi medicinal plants [5, 9–11]. In this study, the plant species were selected either from ethnobotanical or chemotaxonomical data [12]. It is noteworthy to mention that to the best of our knowledge, this study represents the first report on antileishmanial activity for some of the tested plants. The aim of this study was to investigate the antileishmanial activity of the 16 methanol extracts from 13 selected plant species from Albaha region, against* Leishmania amazonensis*.

## 2. Material and Methods

### 2.1. Plant Materials

The plant material was collected in March-April 2014 from different locations in Albahah town and outskirts of Albaha, except myrrh oleo gum resins that were purchased from the herbal shops under the name (Somali Myrrha) (Table 1). The plants were taxonomically identified at the Faculty of Science, Department of Botany, Aden University, Yemen. Voucher specimens of the plant material are deposited at the Pharmacognosy Department, Faculty of Clinical Pharmacy, Albaha University, Saudi Arabia.

### 2.2. Preparation of Extracts

Air-dried and powdered plant material (10 g) was extracted under shaking at room temperature with MeOH (4 × 100 mL). The obtained extracts were filtered and evaporated to dryness* in vacuo* at 40°C. The yields of each dried extract were calculated in %. The resulting dried crude extracts were stored at 4°C. For testing, the extracts were dissolved in dimethylsulfoxide (DMSO) at 20 mg/mL.

### 2.3. Antileishmanial and Cytotoxicity Assays

#### 2.3.1. Parasite Cultures

Strain MHOM/77BR/LTB0016 of* L. amazonensis*, provided by Immunology Department at Oswaldo Cruz Foundation (FIOCRUZ) from Brazil, was used. Parasites were routinely isolated from normal BALB/c mice lesions and maintained as promastigotes at 26°C in Schneider's medium (SIGMA, St. Louis, MO, USA) containing 10% heat-inactivated fetal bovine serum (HFBS) (SIGMA, St. Louis, MO, USA) and corresponding antibiotics (100 *μ*g of streptomycin/mL, 100 U penicillin/mL). Parasites were not used after the tenth passage to carry out* in vitro* experiments.

#### 2.3.2. Antipromastigote Assay

Fifty microliters of Schneider's medium with HFBS and antibiotics was distributed in each well of a 96-well plate. In the first one, additional 48 *μ*L of medium was added as well as 2 *μ*L of extracts. Then, five dilutions 1 : 2 were carried out taking 50 *μ*L each time to test concentrations of the extracts ranging from 12.5 to 200 *μ*g/mL and a final volume was completed to 100 *μ*L after addition of 50 *μ*L of parasites at 4 × 10^5^ promastigotes/mL in logarithmic phase. Also, parasites treated with DMSO or pentamidine (Richet, Buenos Aires, Argentina) were also included as controls. Plates were sealed with parafilm and incubated at 26°C during 72 hours. Then, 20 *μ*L of a solution of 3-[4,5-dimethylthiazol-2-yl]-2,5-diphenyltetrazolium bromide (MTT) (SIGMA, St. Louis, MO, USA) at 5 mg/mL prepared and filtered in the moment of use was added in each well. An additional incubation of 4 hours was performed, the supernatant was eliminated, and the formazan crystals were dissolved with 100 *μ*L of DMSO. The optical density was determined using a spectrophotometer (Sirio S Reader, 2.4-0, Italy), at a test wavelength of 560 nm, using 630 nm as reference wavelength [13, 14]. The median inhibitory concentration (IC_50_) was obtained from linear dose-response curves fit to data by means of the linear equation model. The evaluations were performed in triplicate and the results are expressed as mean ± standard deviation.

#### 2.3.3. Cytotoxicity Assay

The cytotoxic median concentration (CC_50_) of the extracts was also determined on normal peritoneal macrophages from normal BALB/c mice [13]. Resident macrophages were collected from peritoneal cavities of animals in ice RPMI 1640 medium (SIGMA, St. Louis, Mo, USA) supplemented with antibiotics. Suspension was seeded at 30000 cells/well and incubated for 2 hours at 37°C in 5% CO_2_. Nonadherent cells were removed by washing and 50 *μ*L of medium complemented with 10% of HFBS and antibiotics was added. Additional 48 *μ*L was distributed in first well along with 2 *μ*L of tested extracts. Plant extracts were tested at 5 concentrations (200, 100, 50, 25 and 12.5 *μ*g/mL) to establish a full dose-titration and determination of the IC_50_ (inhibitory concentration 50%). The plate was incubated under the same conditions during 72 hours. Macrophages treated with DMSO or pentamidine were included as controls. Cells viability was determined using the colorimetric assay with MTT as previously described, where 15 *μ*L was added to each well, and CC_50_ was obtained from dose-response curves fit to data by means of the linear equation model. The evaluations were performed in triplicate and the results are expressed as mean ± standard deviation.

Then, the selectivity index (SI) ratio was obtained from calculation: CC_50_ for macrophage/IC_50_ for promastigotes [15]. Extracts with SI ≥ 5 or with IC_50_ < 12.5 *μ*g/mL against promastigotes were selected for further experiments.

#### 2.3.4. Antiamastigote Assay

The peritoneal macrophages were obtained as previously described and plated at 10^6^/mL in 24-Well Lab-Tek (Costar, USA). Nonadherent cells were removed by washing after incubation of 2 hours at 37°C in 5% CO_2_. Macrophages were then infected with stationary-phase of* L. amazonensis* promastigotes at 4 : 1 parasite/macrophage ratio and incubated again during 4 hours at the same conditions. Free parasites were removed by washing and 1000 *μ*L of RPMI completed medium was added in each well. In the first well, additional 990 *μ*L of medium and 10 *μ*L of the tested extracts were added and four dilutions 1 : 2 were carried out taking 1000 *μ*L each time, testing final concentrations between 12.5 and 100 *μ*g/mL. The plate was incubated at the same conditions for 48 hours [16]. Then, monolayer was fixed in absolute methanol, stained with Giemsa, and examined under light microscopy. Number of intracellular amastigotes and percent of infected macrophage were determined by counting 25 macrophages per sample, and the results were expressed as percent of reduction of the infection rate (obtained by multiplying the percentage of infected macrophages by the number of amastigotes per infected macrophages) in comparison to controls [15]. IC_50_ value was determined from the concentration-response linear curves. The evaluations were performed in triplicate and the results were expressed as mean ± standard deviation.

## 3. Results and Discussion

Sixteen methanolic extracts of thirteen plant species belonging to ten families gathered from Albaha region were investigated for their* in vitro* antileishmanial activity. Table 1 gives the names of the plants, family, parts investigated, voucher specimen numbers, local names, yields in %, and their traditional uses. Results of antileishmanial activity, cytotoxic effects on peritoneal macrophage from BALB/c mice, and selectivity of methanolic extracts studied are summarized in Table 2. Out of the 16 methanol extracts tested against promastigote forms of* L. amazonensis*, three extracts showed promising activity with potent leishmanicidal activities and good selectivity indices:* A. biebersteinii* flower (IC_50_ = 26.9 *μ*g/mL; SI = 5),* E. helioscopia* leaf (IC_50_ < 12.5 *μ*g/mL; SI = 8), and* S. incanum* leaf (IC_50_ = 18.0 *μ*g/mL; SI = 8). In the case of extracts from* Myrrh* resin,* W. somnifera* and aerial parts of* P. crispa* also showed good antileishmanial activity but a low SI was observed. The rest of extracts tested were either inactive or nonspecific due to high cytotoxicity against normal peritoneal macrophages from BALB/c mice (Table 2). The promising extracts were further tested against intracellular amastigotes of* L. amazonensis*, and four of these extracts were active (Table 2); in particular the extract of flowers from* A. biebersteinii* displayed the most promising activity.

Phytochemical studies of the genus* Achillea* have revealed the presence of a number of phytochemicals that exhibited antileishmanial activity. Santos et al. 2010 [17] reported the antileishmanial activity of an essential oil from the leaves and flowers of* A. millefolium*. Therefore, the antileishmanial activity of* A. biebersteinii* may be attributed either to the components of the essential oil [17] or alkamides that have been isolated from* Achillea* species and possess antileishmanial activity [18].

However,* E. helioscopia* has been the subject of abundant phytochemical and biological investigations, but there are no reports regarding its antileishmanial activity [19]. The plant is used traditionally in Saudi Arabia for removing warts and orally to dispel worms [20]. Several compounds have been isolated from* E. helioscopia* including jatrophone type diterpenoids and the flavones quercetin, and may be responsible for the leishmanicidal activity of the plant extract [21]. In agreement partially with our results, Duarte et al. reported antileishmanial activity for the stilbene piceatannol isolated from* Euphorbia lagascae* against promastigotes of* L. donovani*,* L. infantum,* and* L. major* with moderate activity [22]. Several triterpenoids isolated from* Euphorbia resinifera* and* Euphorbia officinarum* were found to possess antileishmanial activity [23].

Interestingly, antileishmanial activity was observed for* S. incanum* leaves. A number of* Solanum* species with antileishmanial activity have been reported [24–28]. It was found that the extract of* S. torvum* inhibited the proliferation of promastigotes of* L. donovani* [24]. The fruits of* S. stramonifolium* were shown to have marginal activity against amastigotes of* L. amazonensis* [25]. This activity may be attributed to the steroid derivative such as cilistol-A or steroidal alkaloids which form the main compounds in* Solanum* species [26, 27]. The oleo gum resins (myrrh) of* Commiphora* species have long been used for health problems such as stomachache, colds, wounds, malaria, fever and as an antiseptic and against skin infections [28]. Previous studies on the* Commiphora* genus reported no antileishmanial activity of* C. parvifolia* and* C. ornifolia* [29]. This is the first report on the antileishmanial activity of myrrh oleo gum resins. Volatile oil of different* Commiphora* species was found to be rich in beta-elemene, alpha-copaene, alpha-humulene, beta-selinene, and germacrene B. Commiphora oil rich in alpha-humulene may be responsible for antileishmanial activity [28, 30]. Antiplasmodial activities of* C. opobalsamum* and* C. schimperi* were previously reported [31].

*W. somnifera* fruits and leaf (IC_50_ of 20.3 and 29.5 *μ*g/mL) exhibited, in the present study, more remarkable but nonselective antileishmanial activity than that reported previously and collected from India (IC_50_: 63.0 *μ*g/mL) [8]. However, similar activity with an extract collected from Oman was reported (IC_50_: 22.1 *μ*g/mL) [32]. The difference in the activity may be explained in the light of the presence and/or quantities of bioactive compounds in plants that are influenced by several factors including seasons, environment, plant-part used, intraspecies variations, and plant age. It was found that withaferin A, the inhibitor of protein kinase C, is responsible mainly for the higher activity of the plant [8, 33].

In our study,* P. crispa* extract exhibited more potent antileishmanial activity compared to the* P. crispa* extract reported in literature [34].* P. crispa* species were found to possess antileishmanial activity.* P. gnaphalodes* essential oil and extract have good leishmanicidal effects and it seems that the leishmanicidal activity has been mostly related to the terpenoid constituents of the plant [35].

## 4. Conclusion

In conclusion, the evaluation of antileishmanial activity of 16 extracts from 13 plants of Albaha region against* L. amazonensis* has been reported for the first time and revealed that four extracts were found to have promising antileishmanial phytochemical constituents. In particular, the extract of* A. biebersteinii* was the most promising extract and should be further investigated using bioactivity-guided isolation of the active constituents. Moreover, the evaluation of antileishmanial activity confirms the ethnobotanical uses of certain plant extracts such as myrrh oleo gum resins and* S. incanum*.

## Figures and Tables

**Table 1:** Selected plants studied, ethnobotanical information and characteristics.

Species	Plant family (voucher specimen no)	Part tested^a^ (yield in %)	Local name	Traditional uses
*Achillea biebersteinii *Afan.	Asteraceae (CP-101)	Fl (1.5)	Thafra	Antispasmodic and for^1^ kidney inflammation

*Achillea biebersteinii *Afan.	(CP-101)	L (2.6)	Thafra	As antispasmodic and for kidney inflammation

*Calotropis procera* (Aiton) W.T.Aiton	Asclepiadaceae (CP-091)	L (4.2)	Alashur	For treating leprosy and filariasis

*Chenopodium murale *L.	Amaranthaceae (CP-081)	F (7.3)	Jkheara	Leishmaniasis^1^

*Commiphora myrrha * L.	Burseraceae (CP-071)	Resin (70)	Somali mir	Gum used for treating leishmaniasis^2^

*Dodonaea viscosa *Jacq.	Sapindaceae (CP-061)	L (3.5)	Shath	For treating chronic ulcer, burns, leishmaniasis^2^

*Euphorbia helioscopia* L.	Euphorbiaceae (CP-051)	AP (4.2)	Al-dehin	Antiseptic

*Lavandula dentata* L.	Lamiaceae (CP-041)	AP (2.9)	Al-shiah	As antispasmodic, antiseptic when the leaves chewed^1^

*Pulicaria crispa *SCH.BIP	Asteraceae (CP-102)	AP (3.1)	Arararabi	Antimalarial, stomach disorders^2^

*Punica granatum* L.	Punicaceae (CP-011)	Fl (2.5)	Al-roman	Anthelmintic, antiseptic^2^

*Ruta chalepensis *L.	Rutaceae (CP-121)	L (5.2)	Al-shathab	Antimicrobial^2^

*Solanum incanum *L.	Solanaceae (CP-131)	F (7.6)	Al-hadak	Antiseptic^2^

*Solanum incanum *L.	Solanaceae (CP-132)	L (3.9)	Al-hadak	Leaves as dressing for healing wounds, paste of fruits for treating leishmaniasis^2^

*Verbesina encelioides* (Cav.) Benth & Hook. f. ex A. Gray	Asteraceae (CP-021)	L (3.7)	Safeara	Wounds, skin diseases^2^

*Withania somnifera* (L.) Dunal	Solanaceae (CP-011)	F (4.6)	Alobeb	Chronic dermatitis^2^

*Withania somnifera *	Solanaceae (CP-011)	L (8.5)	Alobeb	

^a^AP, aerial parts; F: fruits; L: leaves; Re: resins; Fl: flowers: ^1^most information obtained from reference [12] and ^2^interviewing with local people.

**Table 2:** Activity and cytotoxicity of plant extracts against *L*. *amazonensis* and peritoneal macrophage from BALB/c mice, respectively.

Species (part tested^a^)	IC_50_^b^ ± SD^c^ (*μ*g/mL) Promastigotes	CC_50_^d^ ± SD (*μ*g/mL) Macrophage	SI^e^	IC_50_ ± SD (*μ*g/mL) Amastigotes
***A***. ***biebersteinii *(Fl)**	**26.9 ± 2.9**	**131.3 ± 6.1**	**5**	**13.6 ± 0.6**
*A*. *biebersteinii* (L)	>200	—	—	—
*C*. *procera* (L)	>200	—	—	—
*C*. *murale* (F)	>200	—	—	—
*Myrrha *resin (Re)	17.5 ± 0.01	49.8 ± 2.0	3	—
*D*. *viscosa* (L)	>200	—	—	—
***E***. ***helioscopia *(AP)**	**<12.5**	**82.5 ± 4.4**	**7**	**31.1 ± 3.9**
*L*. *dentata* (AP)	>200	—	—	—
*P*. *crispa* (AP)	23.5 ± 2.5	79.0 ± 7.3	3	—
*P*. *granatum* (Fl)	>200	—	—	—
*R*. *chalepensis* (L)	>200	—	—	—
*S*. *incanum* (F)	37.8 ± 2.7	174.6 ± 6.9	5	24.4 ± 3.0
*S*. *incanum* (L)	18.0 ± 2.8	149.2 ± 3.8	8	31.0 ± 3.8
*V*. *encelioides* (L)	>200	—	—	—
*W*. *somnifera* (F)	29.5 ± 1.0	84.9 ± 4.7	3	—
*W*. *somnifera* (L)	20.3 ± 6.8	<12.5	0	—

^a^AP, aerial parts; F: fruits; L: leaves; Re: resins; Fl: flowers.

^b^IC_50_: Concentration of extract causing 50% growth inhibition.

^c^SD: Standard deviation.

^d^CC_50_: Concentration of extract causing 50% of mortality of peritoneal macrophage from BALB/c.

^e^SI: Selectivity index. —: Not done.
